# The role of academic self‐concepts, parent expectations and teacher–student interactions in socio‐economic gaps in educational attainment

**DOI:** 10.1111/bjep.70025

**Published:** 2025-09-05

**Authors:** Caoimhe Dempsey, Selina McCoy

**Affiliations:** ^1^ School of Applied Psychology University College Cork Cork Ireland; ^2^ The Economic and Social Research Institute Dublin Ireland; ^3^ Department of Sociology Trinity College Dublin Dublin Ireland

**Keywords:** academic self‐concept, educational attainment, parent expectations, socio‐economic differences, teacher‐student interactions

## Abstract

**Background:**

Low socio‐economic status (SES) is a persistent risk factor for educational attainment. Parent expectations and student's academic self‐concepts contribute to this link; however, few studies have examined how changes in these constructs over time contribute to SES gaps in attainment and how teachers may buffer against the consequences of these changes.

**Aims:**

We examine SES differences in (a) changes in parent expectations and academic self‐concepts from primary to secondary school and (b) interactions between teacher–student interaction quality and parent expectations to uncover the role these processes play in SES differences in attainment.

**Sample and Methods:**

Using the Growing Up in Ireland cohort (*N* = 5190), children reported on their academic self‐concepts (9 and 13 years); quality of teacher interactions (13 years); and third‐level attainment (20 years). Primary caregivers reported on their educational expectations and SES (9 and 13 years).

**Results:**

From 9 to 13 years, children from low SES families experience a steeper drop in academic self‐concept and no rise in their parents' expectations compared with their high SES peers. Both initial levels and changes in children's self‐concepts and parents' expectations predicted attainment, with parents' expectations a stronger influence in low SES families. Teacher–child interactions at age 13 moderated the effect of parents' expectations on attainment; however, this was cumulative for low SES children and compensatory for high SES children.

**Conclusions:**

We discuss how findings regarding these developmental processes can be used in school policy and practices aimed at addressing SES differences in educational attainment.

## INTRODUCTION

Children from low SES families are persistently less likely to advance into third‐level education, both in Ireland (Carroll et al., 2022) and internationally (Burger & Mortimer, 2021). The COVID‐19 pandemic has further widened socio‐economic differences in educational attainment (De Witte & François, 2023), as students from low SES families were less likely to have adequate internet connection and devices for online learning, a quiet place to study or a parent at home to help with schoolwork during school closures (Murray, 2022). As such, there is a continued need to examine mechanisms contributing to socio‐economic disparities in educational attainment. The current study addresses this by examining how children's academic self‐concepts, parent expectations and teacher–student relationship quality are linked with their educational attainment. Child and parent data from a national birth cohort study, Growing Up in Ireland, is used at three timepoints: 9, 13 and 20 years, and comparisons are made between families by their exposure to economic vulnerability. First, changes in academic self‐concepts and parent expectations are measured during the transition from primary to secondary school, a critical period in the development of these constructs. Second, an interaction is tested between parent expectations and teacher–student relationship quality on educational attainment. Expanding the developmental and contextual scope of this work, educational attainment is examined at third level, namely whether and at what level (e.g., vocational training, diploma or university degree) students continue their education post‐secondary school.

### Theoretical framework

This study draws on situated expectancy‐value theory (SEVT), which posits that academic performance and achievement‐related choices are influenced by one's expectations for success and self‐concept of ability (Eccles & Wigfield, 2020). Self‐concept is defined as one's perceptions of their competence or ability within a domain, including academic self‐concept (Eccles & Wigfield, 1995; Wigfield & Eccles, 2000). SEVT posits that perceptions of others' attitudes and expectations, such as parents and teachers, contribute to the development of children's self‐concepts (Eccles & Wigfield, 2020, 2024). SEVT posits that the associations between self‐concepts and the perceptions of socializers can be bidirectional and their predictive role in individual differences in achievement changes over time (Eccles & Wigfield, 2020, 2024). Empirical support for the model includes significant changes documented in academic self‐concepts across the school years (e.g., Musu‐Gillette et al., 2015), and links between parental beliefs and behaviours and changes in children's self‐concepts and achievement‐related choices (Simpkins et al., 2015). Recent developments in the theory have placed greater emphasis on the situated nature of these processes within social, economic and cultural context (hence, the recent rename from expectancy‐value theory to situated expectancy‐value theory; Eccles & Wigfield, 2020). Based on this theoretical framework, the current study examines (a) bidirectional associations in changes in academic self‐concepts and parents' expectations and (b) subsequent associations with educational attainment. Contributing to the recent emphasis on context within the theory, these patterns are compared across high and low SES families to understand potential patterns contributing to socio‐economic inequality in education.

While SEVT incorporates the role of teacher beliefs and behaviours, and increasing teacher–student relationship quality (Eccles & Wigfield, 2024), the theory does not account for the interacting influence of parent and teacher factors. This perspective is particularly important when considering socio‐economic differences in developmental outcomes, as inequality operates through cumulative risk (Ainsworth et al., 2015) and developmental cascade models (Dishion & Patterson, 2016). As such, our hypotheses based on SEVT are complemented by adopting a wider developmental systems lens in this paper. Developmental systems theory emphasizes how multiple important adults influence long‐term development for children through both spillover and compensatory pathways (Lerner, 2006; Masten & Cicchetti, 2016). In this study, the cumulative importance of parent and teacher factors is examined to further elucidate socio‐economic disparities in educational attainment and identify potential pathways for promoting educational equality. This approach provides a novel lens for research drawing on SEVT and provides greater insights for practitioners and policymakers regarding the role of teachers in combatting socio‐economic inequality in education.

### Socio‐economic status and educational attainment

Low socio‐economic status (SES) can be conceptualized as a set of adverse circumstances that serve as barriers to accessing economic, social and cultural resources (Duncan & Magnuson, 2012). Studies have used various indicators of low SES, such as low family income, parental education or parental joblessness and most draw upon multiple indicators (Crampton & Hall, 2017). Official definitions of poverty include absolute poverty, where people lack the necessities for survival; relative poverty, where people's income and standard of living are lower than the country in which they live and at risk of poverty, where people are living below the relative money poverty threshold of 60% of the median equivalized household income (EAPN, 2018). The current study uses a measure of relative poverty that accounts for income, material deprivation and family economic stress (Whelan et al., 2015). It is widely documented that low SES is associated with lower academic attainment (Joseph et al., 2024; Sammons et al., 2008), through home resources, social capital and parental education and expectation (Eccles, 1993; Guo et al., 2015). The association between SES and academic attainment emerges early in life (Sammons et al., 2004), persists through adolescence (García & Weiss, 2017) and leads to reduced job prospects in adulthood (Duncan et al., 2010). Moreover, while SES has a strong direct effect on academic ability in the early years, this effect becomes indirect (but larger) by middle childhood (Crampton & Hall, 2017), indicating ‘developmental internalization’ (Sammons et al., 2013). By third level, low SES children are less likely than their more affluent peers to attend third‐level education, and when they do, they are less likely to graduate (Bjorklund‐Young, 2016).

### Children's academic self‐concepts

Academic self‐concepts show reciprocal positive links with academic outcomes (Demetriou et al., 2020; Dempsey et al., 2023; Marsh & Martin, 2011). For example, students with higher math self‐concepts at school are more likely to pursue math‐intensive courses in third‐level education (Umarji et al., 2018). Several studies have identified academic self‐concept as a mechanism contributing to SES differences in educational outcomes, with low SES students more vulnerable to poorer academic self‐concepts (Crampton & Hall, 2017) which in turn contribute to differences in attainment (Guo et al., 2015; Kriegbaum & Spinath, 2016). A study of 396 UK students at the end of secondary school showed that students from households with a higher number of possessions performed better in their exit examinations (A levels), and this was partially explained by differences in students' expectations of success (Brown & Putwain, 2022).

However, academic self‐concepts are not stable over time. In general, academic self‐concepts usually start very positive (Dempsey et al., 2023) and become increasingly negative throughout school (Aunola et al., 2002; Jacobs et al., 2002). A particular drop in self‐concept is documented when children move from primary to secondary school (Coelho et al., 2017), and declines over this transition predict lower scores in math and reported wellbeing at a five‐year follow‐up (Ramos & Verschueren, 2024). Furthermore, while this decline from primary to secondary school is broadly universal (Orth et al., 2021), the decline is steeper for low SES students (Postigo et al., 2022). This aligns with studies showing that low SES students are more likely to experience difficulties adjusting from primary to secondary school more broadly (Gutman & Midgley, 2000; Smyth & Privalko, 2023). As such, targeting academic self‐concepts during the transition from primary to secondary school may be particularly salient for mitigating against widening of socio‐economic gaps in educational outcomes.

### Parental educational expectations

SEVT posits that children's self‐concepts develop in interaction with others and are significantly shaped by the beliefs, expectations and behaviours of socializers, such as parents (Eccles & Wigfield, 2020). The beliefs and expectations from parents may contribute to socio‐economic differences in self‐concept and in turn, educational attainment. Parent expectations have a well‐documented association with children's academic outcomes and self‐concepts (Pinquart & Ebeling, 2020), and high SES families usually hold higher expectations for their children's education, even when controlling for children's academic ability (e.g., Koshy et al., 2019; Michael & Kyriakides, 2023; Smyth, 2020). In turn, children from higher SES families typically report more positive academic self‐concepts (Crampton & Hall, 2017) and are more likely to attend university, even if their cognitive abilities are lower (Paulus et al., 2021).

While initial studies focused on parent‐driven effects, the association between parent expectations and children's self‐concept appears to be transactional, with parent expectations also responsive to their child's academic self‐concepts (Briley et al., 2014). Examining these transactional associations during the transition from primary to secondary school, using data at 9, 12 and 15 years, reciprocal associations of equal strength were found between parent aspirations (capturing hopes rather than expectations) and children's academic self‐concepts (Buchmann et al., 2022). This differs somewhat from the pattern of associations in expectations during primary school, where only parents' beliefs predicted changes in their child's self‐concepts, but not vice versa (Simpkins et al., 2015). Furthermore, the nature of these associations may differ by family SES. Findings from the UK's Millennium Cohort Study showed that reciprocal associations between achievement and aspirations (not expectations) at 7, 11 and 14 years were only significant among high and medium SES groups (Park et al., 2024). Despite having relatively high aspirations, these did not significantly predict achievement at any age point for low SES students (Park et al., 2024). Together, these findings indicate that while parents are likely an important contributor to changing self‐concepts during the transition to secondary school, more evidence is needed on the direction of associations during this period, particularly in relation to low SES students and subsequent links with educational attainment. The current study aims to address this gap and provide evidence for efforts to address socio‐economic inequality.

### Moderating role of teacher–child interactions

Aside from family influences, research has shown that positive teacher–child interactions also support children's academic self‐concepts (Leflot et al., 2010; Verschueren & Koomen, 2012) and achievement (Roorda et al., 2017). Indeed, interpersonal relationships have been identified as particularly important in addressing declines in students' motivational factors during the transition to secondary school (Martins et al., 2021; Wang et al., 2019). For example, growth curve analysis tracking 1011 US students at four timepoints during the transition from primary to secondary school showed that parental influences contributed to initial levels of student engagement, whereas teacher involvement contributed to changes in student engagement (Rickert & Skinner, 2024). In line with developmental systems theory (Masten & Cicchetti, 2016), children engage in multiple interpersonal relationships, and educational outcomes are best considered in the context of the collective contributions of multiple socializers across home and school contexts (Martin & Dowson, 2009). This approach has previously shown that positive teacher–child relationships can buffer against the negative associations between low‐quality family relationships and academic outcomes (Dempsey et al., 2024; Nauman et al., 2023).

Findings are mixed regarding how the moderating effect of teachers may differ by family SES. In a sample of 1,077 US students tracked from 11 to 15 years, teacher–child relationships only acted as a moderator of maternal relational adversity on language development in middle‐ and upper class, but not lower class, families (Nauman et al., 2023). By contrast, in a sample of 19,465 Australian students tracked across the transition from primary to secondary school, the predictive paths of perceived teacher support on subsequent motivation, engagement and attainment were largely invariant across student attributes, suggesting teaching support yields positive effects across students (Martin et al., 2024). Using data from 13,100 US students to examine predictors of STEM career expectations, students with non‐college educated parents were significantly more likely to change from STEM to non‐STEM career expectations during adolescence; however, all students were more likely to maintain STEM career expectations through high school if they received support from teachers (Starr et al., 2022). Adding to this mixed literature, the current study tests socio‐economic differences in interactions between teacher–child relationships and parent expectations for students' educational attainment.

### Irish context

Ireland has a centralized education system with a strong emphasis on terminal standardized examinations and a high entry into third‐level education (OECD, 2024). While Ireland ranks highly in international achievement studies such as Trends in International Mathematics and Science Study and Programme for International Student Assessment (OECD, 2023; Perkins & Clerkin, 2020), there are persistent socio‐economic gaps in achievement and attainment at both primary and secondary school levels (OECD, 2024). At the primary level, this gap is similar to the average gap internationally as reported in PIRLS 2021 (88.5 vs. 86.1 points; Delaney et al., 2023) and TIMSS 2019 (Mullis et al., 2020). By the secondary level, gaps in Ireland are smaller than international comparisons, with PISA data showing Irish gaps in maths (73.7 points), reading (75.6) and science (78.2) all lower than the OECD averages (93.5, 93.0, 96.2 points, respectively; OECD, 2023). Educational inequality is targeted in Ireland through the Delivering Equality of Opportunity in Schools (DEIS; 2017) programme, which provides support to schools with a high concentration of disadvantage such as additional classroom teaching posts, home school community coordinator posts and school completion programme.

### Current study

The current study aims to identify potential contributors of socio‐economic educational inequality by drawing on SEVT (Eccles & Wigfield, 2020). Based on previous literature and SEVT, we hypothesize that:

- Self‐concepts will drop from primary to secondary school (Coelho et al., 2017), and low SES students will report lower initial self‐concepts and parent expectations, and steeper drops in self‐concepts from primary to secondary school (Postigo et al., 2022).
- Changes in self‐concepts and parent expectations will be bidirectionally related (Buchmann et al., 2022; Simpkins et al., 2015) and longitudinally associated with educational attainment for all students (Eccles & Wigfield, 2020; Ramos & Verschueren, 2024).
- Teacher–student interactions will moderate the association between parent expectations and educational attainment, acting as a buffer in the context of low parent expectations (Starr et al., 2022). Given the mixed literature on SES differences in the moderating role of teacher–student relationships (Nauman et al., 2023; Starr et al., 2022), our socio‐economic comparison of this interaction is exploratory.

## METHOD

### Participants

Participants were from Growing Up in Ireland (GUI), a nationally representative cohort study of children in Ireland. The current study uses data when the children were aged 9 (T1; *N* = 8570), 13 (T2; *N* = 7525, 88.9% response rate) and 20 (T3; *N* = 5190, 61% response rate; 48% male). Children were recruited via primary schools, of which 910 primary schools participated in the study, and the within‐school response rate was 57% (Williams et al., 2010). Written consent was obtained from a caregiver and the child before data collection. The GUI study received ethical approval from the research ethics committee within the Department of Children and Youth Affairs. For details of the study design, variables and demographic information, see: https://www.growingup.gov.ie/. During home visits, trained researchers conducted interviews and direct assessments with parents and children. Representative of the general population, families were well educated; 42.1% of primary caregivers held a university degree or higher. Most families were two‐parent (85%) with more than one child (8% only child families). Inter‐wave attrition in GUI was related to family social class, income and parental education (Murphy et al., 2018) and data were statistically re‐weighted using sampling weights representative of the Irish population (Williams et al., 2009).

### Measures

#### Children's academic self‐concept

At T1 and T2, children reported on perceptions of their ability in school, using the Intellectual and School subscale of the second edition Piers‐Harris Self‐Concept Scale (Piers & Herzberg, 2002). The subscale consists of 16 items reflecting participants judgements of their own intellectual abilities and academic performance and their expectations about their achievement (Murray et al., 2011), such as ‘I am smart’ and ‘My classmates in school think I have good ideas’. Participants responses on a binary Yes/No to indicate whether they feel the item applies to them or not were summed to create a total score with higher ratings indicating more positive self‐concepts (T1 *α* = .81; Murray et al., 2011; T2 *α* = .82, Thornton et al., 2016). Previous studies show good test–retest reliability across ages 7–18 and good internal consistency (α ranging from .74 to .81; Piers & Herzberg, 2002).

#### Parental educational expectations

At T1 and T2, primary caregivers responded to a single item question: ‘Taking everything into account, how far do you expect your child to go in his/her education or training?’. Participants chose from six response options: lower secondary school (age 16), upper secondary school (age 18), vocational training, sub‐degree diploma or certificate, undergraduate degree and postgraduate degree.

#### Teacher‐child interaction quality

At T2, children rated their perceived quality of interactions with teachers. Three items were used: ‘You are told by a teacher that your work is good’; ‘You are given out to [reprimanded] by a teacher because your work is untidy or not done on time’; and ‘You are given out to [reprimanded] by a teacher for misbehaving in class’. Items were measured on a four‐point Likert scale (very often/often/a few times/never). The first item was reverse‐coded, and the three items were summed to create a measure of the quality of teacher–child interactions, consistent with previous studies (T2 *α* = .858). This rating of teacher interaction quality has previously been shown to predict educational achievement (McCoy et al., 2014) and early school leaving (Carroll et al., 2022).

#### Third‐level educational attainment

At T3, young people reported on the level of education they pursued after leaving secondary school. Responses were categorized into five options: upper secondary school, vocational training, sub‐degree certificate or diploma, degree at an institute of technology and degree at a university.

#### Family socio‐economic status

The current study uses a measure of economic vulnerability previously developed using the data. Maître et al. (2021) used latent class analysis to identify families who have a high probability of experiencing a distinctive risk profile in relation to three dimensions at a single timepoint: household income quartile position, economic stress and material deprivation. The LCA allocated a probability of belonging to each latent (categorical) class for each family, and modal allocation was used to identify family economic vulnerability based on class membership on the three indicators (Maître et al., 2021). This latent class analysis has been used to operationalize economic vulnerability and multidimensional poverty in previous European (Breen & Moisio, 2003; Moisio, 2004) and Irish research (Watson et al., 2014; Whelan et al., 2015).

The first indicator used the distribution of equivalized disposable household income (net income adjusted for household size and composition) to allocate each family into an income quintile position. The bottom income quintile was used as the proportion for economic vulnerability. The second indicator, economic stress, was based on one question asked to primary caregivers about the difficulty making ends meet, with six possible answers ranging from ‘great difficulty’ to ‘very easily’. Households were considered to experience economic stress when they reported ‘great difficulty’ or ‘difficulty’ making ends meet. This measure is collected in several national and international surveys (e.g., European Quality of Life Surveys; Maître et al., 2021). The third indicator, material deprivation, was based on an Irish measure of basic deprivation that identifies households lacking essential goods and services out of an 11‐item list (e.g., being able to afford two pairs of shoes, having protein rich meals). The measure was used as a continuous indicator (scale of 0–11) within the LCA. Table A1 in the appendix reports the LCA results at 9 and 13 years. Comparing model fit across one, two and three latent classes, Maître et al. (2021) determined the two‐class model categorizing families as economically vulnerable (EV) or not best fit the data (see Appendix for further details).

Following previous work with the variable in this sample (Carroll et al., 2022; Maître et al., 2021) we developed a profile of EV based on exposure over two timepoints, 9 and 13 years. Families were divided into two categories: not EV, which consisted of those who were never EV across the two waves, and EV, which consisted of those who were EV at either 9 years, 13 years or both. Classification into the EV group was heavily impacted by the Irish economy falling into recession, and as such, the direction of flow into and out of the EV group was largely unidirectional, with families entering EV (see Table A1 in the appendix for further details). As such, and considering meaningful interpretation of our hypotheses, separate categories were not created for families who were EV at a single timepoint. It is possible that this, and other poverty transitions between timepoints, could affect the interpretation of our classification.

#### Covariates

Direct assessments of children's reading and maths ability were collected at T1 using standardized national assessments; the Drumcondra Primary Reading Test and Mathematics Tests (Educational Research Centre, 2007). Each test consisted of 40 questions, and children were awarded one mark for each correct answer, and scores on the reading and maths tests were summed to create a general ability score. The Drumcondra Tests correlate well with ability measures such as digit span and letter/number sequencing tasks (Hayes & Stewart, 2016). Children's special educational needs (SEN) status was identified at T1, using parent and teacher reports of whether the child had learning, speech, physical or behavioural difficulties, as well as scores above the clinical cut‐off on the total difficulties measure from the teacher‐report Strengths and Difficulties Questionnaire (SDQ; Goodman, 1997) to create a combined binary measure of either (0) without SEN or (1) with SEN (McCoy et al., 2016). We did not distinguish between types of SEN for this covariate. Primary caregivers reported on child gender at T1.

### Analytic strategy

**FIGURE 1:**
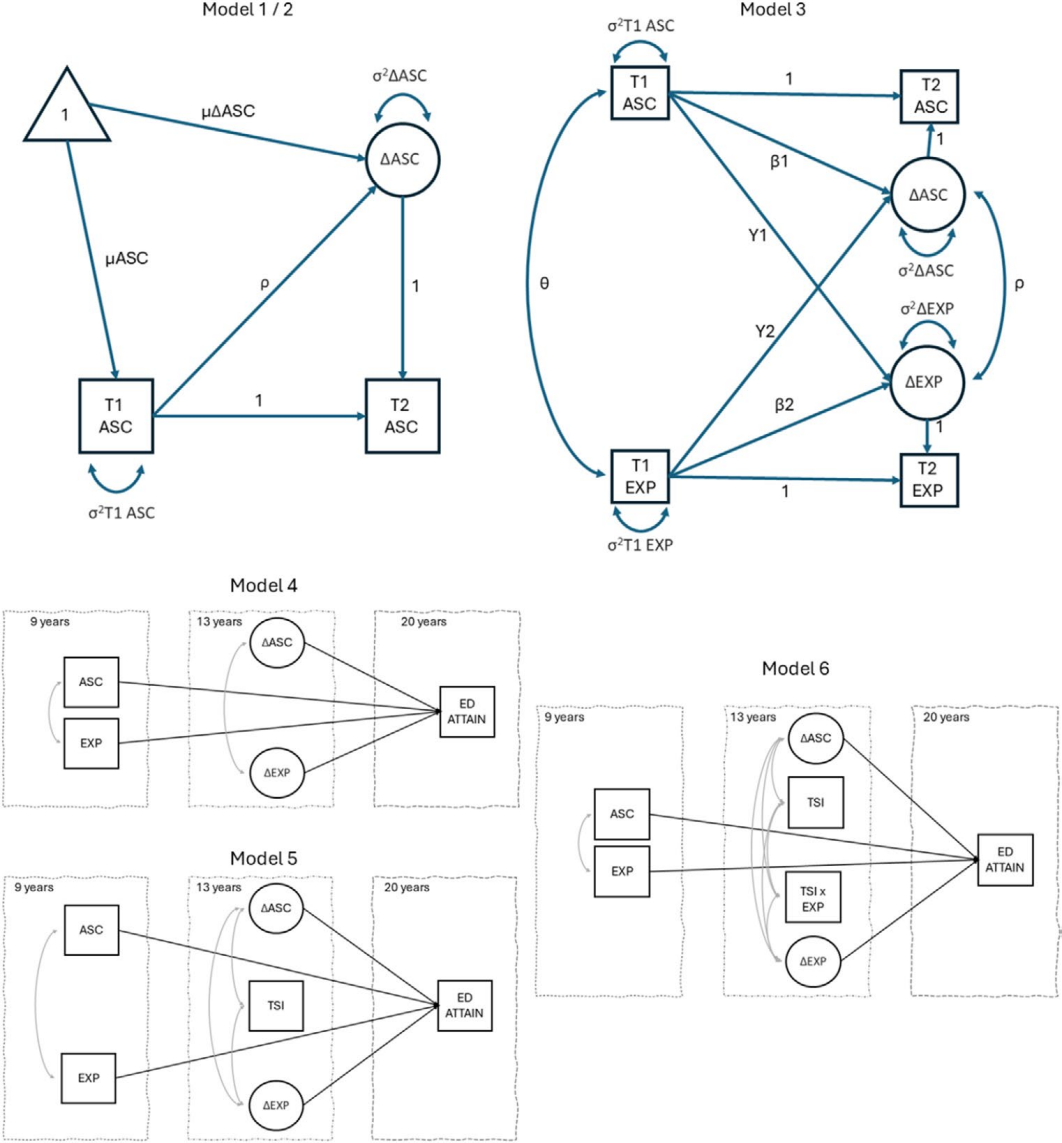
Hypothetical univariate and bivariate change score model and structural equation models. (Model 1) Univariate change score model illustrating the average change in children's academic self‐concepts (ΔASC), variance in change (*σ* ^2^ΔASC) and change depending on the initial measurement (*ρ*). The same model structure was applied to parent expectations (Model 2). (Model 3) Bivariate latent change score model illustrating correlations between academic self‐concepts and parent expectations (EXP) at baseline (*ϑ*), regression between ASC and change in EXP (Y1), regression between EXP and change in ASC (Y2) and co‐occurrence of changes in ASC and EXP (*ρ*). T1 = 9 years, T2 = 13 years. Family EV was introduced at the baseline level. Adapted from Kievit et al. (2018). (Models 4–6) Structural equation models of educational attainment. For ease of presentation, Models 4–6 do not show covariates. ASC, academic self‐concept; ED ATTAIN, educational attainment; EXP, parent expectations; TSI, teacher–student interactions.

We analysed data using the psych, lavaan and ggplot2 packages in R Studio (v4.2.2; R Core Team, 2022). To address missing data, all models were estimated using a full information maximum likelihood (FIML) estimator. The longitudinal sample weight at age 20 was applied to all inferential analyses. In line with previous longitudinal GUI studies, multi‐level modelling was not used as children were not sufficiently clustered by school beyond the first wave of data (e.g., Carroll et al., 2022; Smyth & Privalko, 2023). We applied a maximum likelihood estimator with robust standard errors (MLR) in each of our cross‐lagged panel models to account for the non‐normal distribution of our indicators. First, we specified univariate change score models to estimate the initial levels, means and variances for changes in children's self‐concepts (Figure 1, Model 1) and parents' expectations (Figure 1, Model 2). We then used a bivariate change score model to examine longitudinal associations between self‐concepts and expectations (Figure 1, Model 3) (Kievit et al., 2018). We used a multi‐group procedure to test whether the paths in these models differed across EV and non‐EV families. A Satorra–Bentler scaled *χ* ^2^ difference test (Satorra & Bentler, 2001) was used to test for differences between families categorized as experiencing economic vulnerability (EV) or not EV in these univariate and bivariate models. A significant decrease in model fit after the inclusion of equality constraints suggests the strength of at least one of the constrained paths differs across the two groups. Each model was re‐estimated and grouped by family EV to compare constrained and unconstrained models, and paths were released one by one to find areas of misfit. In all models, we evaluated model fit using three standard criteria: a root mean square error of approximation (RMSEA) of <.08, a comparative fit index (CFI) of >.90 and a Tucker Lewis Index (TLI) of >.90 (Brown, 2015).

Second, to examine associations with educational attainment, we specified a structural equation model that regressed attainment onto initial levels and changes in children's self‐concept and their parent's expectations, as well as our covariates, SEN status and academic ability (Figure 1, Model 4). To examine the moderating role of teacher–child interaction quality, we extended this structural model by additionally regressing educational attainment onto teacher–child interaction quality (Figure 1, Model 5). After presenting the main association of teacher–child interaction quality, we added an interaction term for teacher–child interactions and parent expectations into the model of educational attainment (Figure 1, Model 6). A significant interaction was probed for statistical significance at 1 SD above and below the mean (Aiken et al., 1991). Follow‐up analyses were conducted to plot the simple slopes of the moderated associations. We used a multi‐group procedure to test whether the paths in these models differed across EV and non‐EV families. A Satorra–Bentler scaled *χ* ^2^ difference test (Satorra & Bentler, 2001) was used to test for differences between families categorized as experiencing economic vulnerability (EV) or not EV in these univariate and bivariate models. A significant decrease in model fit after the inclusion of equality constraints suggests the strength of at least one of the constrained paths differs across the two groups. Each model was re‐estimated and grouped by family EV to compare constrained and unconstrained models, and paths were released one by one to find areas of misfit. We evaluated model fit using the same criteria as above.

## RESULTS

### Descriptive statistics and data reduction

Table 1 shows the descriptive statistics for the main study variables.

**TABLE 1:** Descriptive statistics, concurrent and longitudinal correlations for study variables.

		T1		T2		T3		Academic ability T1	Economic vulnerability	SEN status T1
		1	2	3	4	5	6			
1	T1 Academic self‐concept	—	.128***	.298***	.123***	.172***	.157***	.203***	−.08***	−.200***
2	T1 Parental expectation		—	.124***	.459***	.117***	.292***	.339***	−.124***	−.212***
3	T2 Academic self‐concept			—	.202***	.420***	.193***	.214***	−.08***	−.162***
4	T2 Parental expectation				—	.157***	.345***	.364***	−.169***	−.217***
5	T2 Teacher interactions					—	.191***	.129***	−.09***	−.102***
6	T3 Attainment						—	.396***	−.209***	−.218***
	M (SD) or %	12.56 (2.77)	Low Sec 0.21% Upp Sec 6.58% Vocational 4.47% Sub‐degree 9.12% Degree 60.65% Postgrad 28.08%	12.28 (2.99)	Low Sec 0.06% Upp Sec 3.41% Vocational 2.59% Sub‐degree 6.87% Degree 60.65% Postgrad 36.15%	9.78 (1.65)	Upp Sec 10.21% Vocational 16.45% Sub‐degree 6.00% Degree (IT) 16.81% Degree (Uni) 51.52%	66.48 (18.21)	Not EV 79.76% EV 20.23%	No SEN 82.5% SEN 17.5%

Abbreviations: Low Sec, lower secondary school; Upp Sec, upper secondary school.

***p < .001.

### SES differences in children's academic self‐concept and parental expectations from primary to secondary school

The first model examined initial levels, means and variances for changes in academic self‐concepts from 9 to 13 years, and showed good fit to the data (Table 2, Model 1). On average, there was a significant mean decrease in children's self‐concepts from 9 to 13 years, Est. = −.281, SE = .051, *p* < .001, as well as significant inter‐individual differences in the degree of change over time, Est. = 11.697, SE = .290, *p* < .001. There was a significant negative correlation between T1 self‐concept and change score, Std. Est. = −.550, *z* = −1.524, *p* < .0001, such that children with higher initial levels of self‐concept showed the least decrease across the transition. There was a significant reduction in model fit when all paths were constrained to be equal across family economic vulnerability, Δ*χ* ^2^ (1) = 29.939, *p* = .0001. Initial ratings of self‐concept had a stronger correlation with change in self‐concept across the timepoints for non‐EV children, Std. Est. = −.559, *z* = −1.533, *p* < .0001, than EV children, Std. Est. = −.535, *z* = −1.548, *p* < .0001. EV children also report a steeper decrease in self‐concept as indicated by the mean change score, Est. = −.662, SE = .109, *p* < .001, compared with non‐EV children, Est. = −.196, SE = .054, *p* < .001. Further, there were larger inter‐individual differences in the degree of change over time for EV children, Est. = 14.457, SE = .765, *p* < .001, compared with non‐EV children, Est. = 11.032, SE = .309, *p* < .001.

**TABLE 2:** Model fit statistics.

Model	Model description	$\chi^{2}$	df	CFI	TLI	RMSEA
1	CSM academic self‐concept	28.484	3	.940	.920	.076
2	CSM parent's academic expectations	73.815	3	.942	.825	.089
3	Bivariate CSM academic self‐concept and parental expectations	141.815	3	.921	.841	.094
4	Self‐concept and expectations → Attainment	37.713	4	.982	.946	.044
5	Self‐concept, expectations and teacher interactions → Attainment	26.983	4	.988	.965	.035
6	Teacher/parent interaction term → Attainment	263.201	4	.985	.954	.110

Abbreviations: CFI, comparative fit index; CSM, change score model; RMSEA, root mean square error of approximation; TLI, Tucker Lewis Index.

Turning to parental expectations, the model showed moderate fit to the data, exceeding the criteria for CFI but not TLI or RMSEA (Table 2, Model 2). On average, there was a significant mean increase in expectations, Est. = .227, SE = .015, *p* < .001, as well as significant inter‐individual differences in the degree of change over time, Est. = 1.026, SE = .034, *p* < .001. There was a significant negative correlation between T1 expectations and change score, Std. Est. = −.600, *z* = −.620, *p* < .0001, such that parents with higher initial expectations showed the least increase across the transition. There was a significant reduction in model fit when all paths were constrained to be equal across economic vulnerability, Δ*χ* ^2^ (1) = 85.907, *p* = .0001. EV parents showed no increase in expectations as indicated by the mean change score, Est. = −.014, SE = .034, *p* = .681, whereas non‐EV parents showed an increase across timepoints as indicated by the mean change score, Est. = .269, SE = .016, *p* < .001. Changes in expectations among EV parents were also less strongly correlated with initial expectations, Std. Est. = −.541, *z* = −.672, *p* < .0001, than among non‐EV parents, Std. Est. = −.640, *z* = −.624, *p* < .0001.

In Model 3, we estimated a bivariate change score model of parent expectations and academic self‐concepts. The model showed moderate fit to the data, exceeding the criteria for CFI but not TLI or RMSEA (Table 2, Model 3). Parental expectations at T2 were predicted by both initial levels of children's self‐concepts, Std. Est. = .255, SE = .084, *p* < .001, and changes in children's self‐concepts, Std. Est. = .333, SE = .304, *p* < .001. Children's academic self‐concepts at T2 were not predicted by initial levels of parent expectations, Std. Est. = .036, SE = .103, *p* = .148; however, changes in parent expectations did show an effect, Std. Est. = .203, SE = .607, *p* < .001, such that increases in expectations from T1 to T2 were associated with higher self‐concepts at T2. There were small positive associations between changes in self‐concept and changes in expectations, Std. Est. = .098, *z* = .098, *p* < .0001, indicating that increases in self‐concept were positively linked with increases in parental expectations. No additional areas of misfit were found beyond those already identified in the univariate models. Table 2 shows model fit statistics for univariate and bivariate models.

### Children's academic self‐concept and parental expectations as predictors of educational attainment

Next, we specified a structural model of educational attainment to include initial levels and changes in parents' expectations and children's self‐concepts and educational attainment, economic vulnerability, academic ability and SEN status. This model provided a good fit for the data (Table 2, Model 4). Standardized estimates for the model are presented in Table 3. Higher educational attainment was significantly positively related to children's self‐concepts and parents' educational expectations at age 9. Over and above these initial levels, changes in parents' expectations and children's self‐concepts were uniquely associated with educational attainment. Specifically, increases in parents' expectations and increases in children's academic self‐concept across the transition to secondary school were associated with higher levels of educational attainment. There was a significant reduction in model fit when all paths were constrained to be equal across EV, Δ*χ* ^2^ (6) = 52.597, *p* = .0001. Both initial parental expectations and changes in expectations had a stronger role on educational attainment for EV children, initial levels Std. Est. = .297, *z* = .388, *p* < .0001, change scores Std. Est. = .206, *z* = .324, *p* < .0001, compared with their non‐EV counterparts, initial levels Std. Est. = .263, *z* = .388, *p* < .0001, change scores Std. Est. = .173, *z* = .248, *p* < .0001. T1 academic ability was also a stronger predictor of educational attainment for EV children, Std. Est. = .265, *z* = .021, *p* < .0001, than non‐EV children, Std. Est. = .255, *z* = .021, *p* < .0001.

**TABLE 3:** Standardized estimates for models predicting attainment at age 20.

	Model 4		*β*	Model 5		*β*
	Attainment			Attainment		
	*B*	SE		*B*	SE	
Self‐concept initial levels T1	.059	.009	.111***	.020	.001	.250***
Self‐concept changes T1–T2	.036	.007	.084***	.016	.008	.038*
Expectations initial levels T1	.389	.026	.272***	.361	.026	.255***
Expectations changes T1–T2	.262	.026	.180***	.238	.026	.166***
Academic ability T1	.021	.001	.263***	.020	.001	.250***
SEN status T1	−.217	.060	−.057***	−.194	.059	−.051***
Teacher interaction quality T2	—	—	—	.082	.013	.094***

***p < .001, **p < .01, *p < .05.

### Teacher‐child interaction quality as a moderator of parental expectations

**FIGURE 2:**
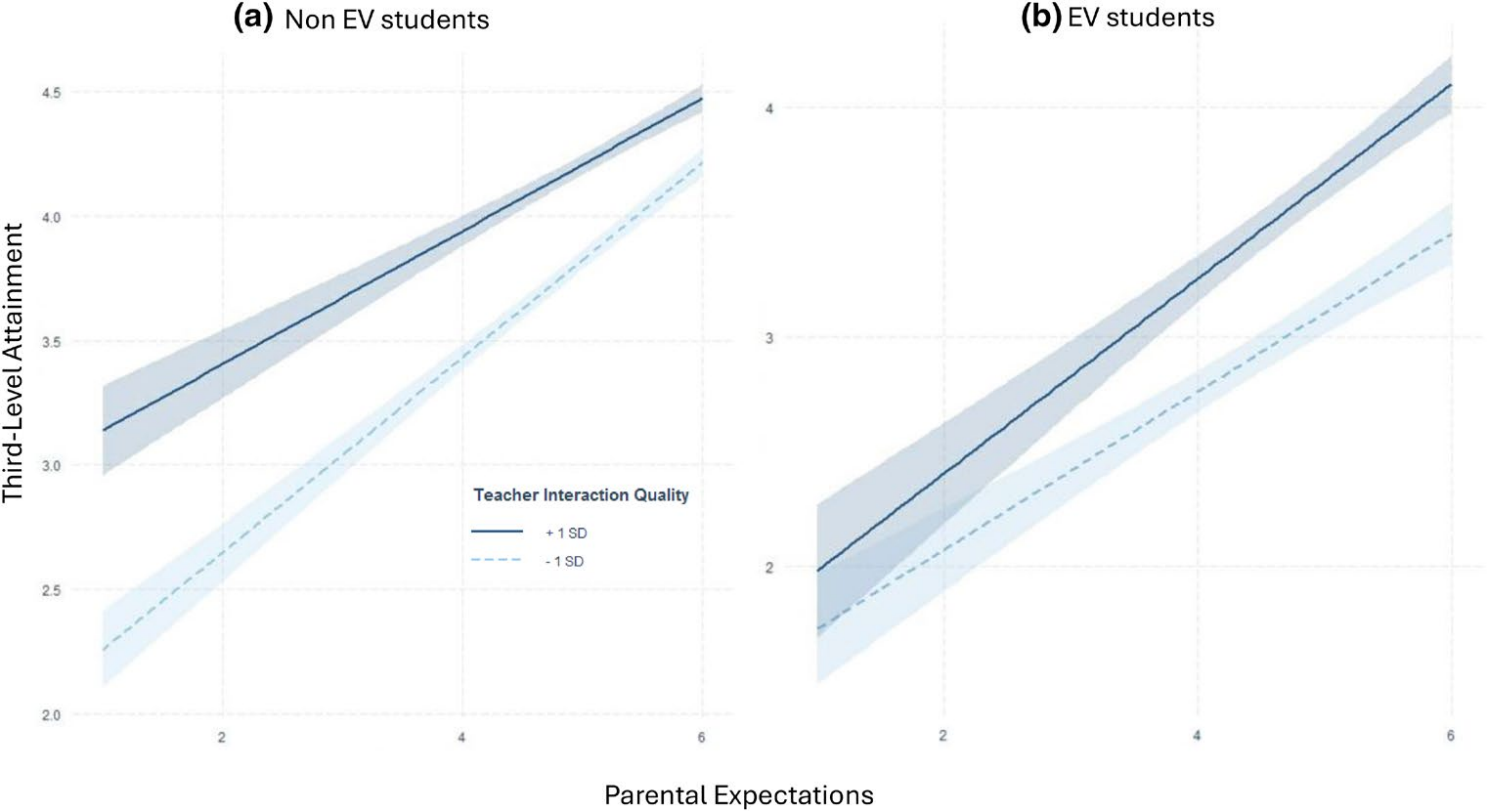
Simple slope plots depicting differences in association between parent's expectations and attainment at +1 SD and −1 SD of teacher interaction quality for (a) non‐economically vulnerable children (*N* = 3772) and (b) economically vulnerable children (*N* = 957).

Finally, we extended the structural model to include a main association of teacher–child interaction quality. The model showed a good fit to the data (Table 2, Model 5). Teacher–child interaction quality was significantly associated with educational attainment, Std. Est. = .094, SE = .013, *p* < .0001 (see Table 3, Model 5). Next, the interaction term was added between teacher–child interactions and parental expectations (Table 2, Model 6). The model showed a good fit to the data (Table 2, Model 6). The interaction term between parents' expectations and teacher interactions had a significant association with educational attainment. In Model 6, there was a significant reduction in model fit when all paths were constrained to be equal across EV, Δ*χ* ^2^ (5) = 59.453, *p* = .0001. Beyond the areas of misfit already reported in Model 4, the interaction term between parent expectations and teacher–child interaction quality had a differing association with educational attainment for EV children, Std. Est. = .154, *z* = .019, *p* < .0001, and non‐EV children, Std. Est. = − .097, *z* = .014, *p* < .001. Figure 2 shows simple slopes of educational attainment and parents' expectations at low and high levels of teacher interaction quality. Positive teacher interactions compensated for lower parental expectations among high SES children (Figure 2a), whereas for children experiencing economic vulnerability (Figure 2b), there was a synergistic role of parents' expectations and high‐quality teacher interactions on educational attainment.

## DISCUSSION

Widened socio‐economic differences in education in the wake of the COVID‐19 pandemic (De Witte & François, 2023) have further emphasized the need for research on socio‐economic educational inequality. Based on situated expectancy‐value theory (Eccles & Wigfield, 2020) and developmental systems theory (Lerner, 2006) and using data from a nationally representative cohort of Irish families, the current study identifies several socio‐economic patterns that contribute to differences in educational attainment. First, children from economically vulnerable (EV) families reported lower levels of academic self‐concept in primary school and a steeper drop in academic self‐concept from primary to secondary school than their peers. Second, unlike their peers, EV parents' expectations do not rise from primary to secondary school. Third, both initial levels and changes in self‐concepts and expectations were independently associated with third‐level attainment, with parent expectations exerting more strongly associated among EV children. Finally, positive teacher–child interactions moderated the association between parent expectations and educational attainment, but this was cumulative for EV children and compensatory for non‐EV children. Below, we discuss these findings in turn, including their utility for educational practice and policy, before considering the limitations of the study.

### Importance of bolstering academic self‐concepts in primary school

Our first hypothesis was supported, with a significant decrease in academic self‐concepts, heightened among EV students, reported from primary to secondary school consistent with previous studies (Postigo et al., 2022; Ramos & Verschueren, 2024). In line with the central tenant of situated expectancy‐value theory (Eccles & Wigfield, 2020), our second hypothesis was also supported, as changes in self‐concept were associated with educational attainment. This finding expands prior subject‐specific evidence on the effects of self‐concepts for third‐level educational attainment (Musu‐Gillette et al., 2015). Contributing to the theory of self‐concept development (Eccles & Wigfield, 2020), children show significant inter‐individual differences in the extent of this decline, with those reporting higher levels of self‐concept at age 9 being more likely to report stable self‐concepts; and these changes have a unique association with third‐level educational attainment evident at a 10‐year follow‐up, particularly among EV children. Our focus on changes during the primary–secondary school transition has important educational practice implications, indicating that bolstering academic self‐concepts *before* starting secondary school could particularly help EV children maintain stable positive academic self‐concepts while adjusting to the many changes experienced in secondary school. In the context of these findings, the shift in educational policy to whole‐child approaches and emphasis on socio‐emotional development seen in Ireland (Department of Education, 2023b) and internationally (CASEL, 2020) is a welcome development and should be further pursued.

### Secondary school gap in parent expectations contributes to EV differences in educational attainment

Contributing to the debate regarding the direction of association between parent expectations and academic self‐concepts, our second hypothesis of bidirectional associations between these constructs was partially supported. Parent expectations were associated with initial levels and changes in academic self‐concepts, whereas self‐concepts were associated with changes, but not initial levels, of parent expectations. This differs from associations between these constructs in primary school, which are largely parent‐driven (Simpkins et al., 2015). This shift over time may reflect changes in cognitive maturity and opportunities to observe ability between parents and students during middle childhood, resulting in parents relying more on feedback from their child by contrast to the parent‐driven effects in early childhood (Simpkins et al., 2015).

Initial levels and changes in parent expectations were associated with educational attainment for all students, in line with our second hypothesis. However, contributing to the recent emphasis on the situated nature of SEVT, the strength of this association was greater among EV students than non‐EV students. This differs from recent analyses of the UK's Millennium Cohort Study, which showed that students' aspirations were associated with achievement in high and medium, but not low, SES students (Park et al., 2024). This is likely to reflect the important difference between expectations and aspirations (wanting to excel in a certain domain; Frenzel et al., 2010). While expectations are largely positively associated with achievement, overly high aspirations that exceed realistic expectations (the aspiration‐expectation gap) may reduce students' academic self‐concept and jeopardize achievement (Marsh et al., 2023; Pekrun, 2021). The documented socio‐economic divergences in parent expectations during the transition to secondary school have important policy implications, particularly considering changes in parent expectations showed an equal magnitude of association with educational attainment as did age 9 reading and maths ability. Mitigating this socio‐economic divergence could be significant in addressing educational inequality. In the Irish context, this highlights the critical role played by home‐school community liaison officers in schools serving low‐income families and the value of initiatives such as the Education Passport that aim to connect parents and primary and secondary school staff (Department of Education, 2017). Internationally, a transition between schools during middle childhood is a common feature of education systems. As such, strategies that engage with parents during this period could have a significant impact for ameliorating socio‐economic differences in long‐term educational trajectories across diverse educational systems.

### Teacher‐child interactions play different roles for low and high SES families

In line with developmental systems theory (Lerner, 2006) and our third hypothesis, teacher–student interaction quality moderated the association between parent expectations and student's educational attainment. Our results extend the scope of the teacher moderating role to parent expectations, previously documented for maternal relationship quality (Dempsey et al., 2024; Nauman et al., 2023). Acknowledging the role of cumulative risk on child development (Evans et al., 2013), we explored whether the moderating role of teacher–student interactions differed depending on whether parent expectations were in the context of family EV or not. Previous literature has shown mixed results: some studies indicate positive teacher–child relationships only buffer against maternal relational adversity in middle and high SES, but not low SES families (Nauman et al., 2023); others indicate teacher support has a universal association with achievement (Martin et al., 2024) and STEM career expectations (Starr et al., 2022) regardless of student attributes or parent education. Adding to this growing literature, the current results indicate that positive teacher–student interactions only benefit EV students whose parents also hold high expectations, that is, parents and teachers provide cumulative positive contributions to educational attainment. This pattern tentatively aligns with the cumulative risk perspective (Evans et al., 2013) and previous research (Nauman et al., 2023) indicating academic resilience is limited in the face of multiple risk factors (in this case, economic vulnerability and low parent expectations). As such, efforts to target socio‐economic educational inequality should take a dual site approach spanning home and school, such as home school community liaison officers in Ireland (Department of Education, 2017). In contrast, for non‐EV children, teacher–child interaction quality appears to play a compensatory role in the context of low parental expectations. This aligns with previous findings of the benefit of a close relationship with *either* mothers or teachers for children's achievement and behavioural adjustment in primary school (Dempsey et al., 2024). Identifying the needs of students and tailoring support efforts based on this evidence could improve the effectiveness of school practices both in Ireland and internationally. In the Irish context, where relatively limited financial barriers to education exist, a community‐based approach to mitigating socio‐economic differences appears to have significant potential.

### Limitations

This study draws on a nationally representative sample with data collected at 9, 13 and 20 years and considers the role of multiple socializers to account for the development of socio‐economic educational inequality. However, the study has several limitations. Family economic vulnerability was categorized as either never experiencing economic vulnerability or experiencing economic vulnerability at either 9 years, 13 years or both. This categorization was used due to the small number of families exiting vulnerability from 9 to 13 years and for ease of model comparisons. However, significant qualitative differences may exist in the families who experienced vulnerability at 9 (before the economic recession in Ireland), the large number of families who entered vulnerability by 13, and those who exited the vulnerability threshold. Future work examining these groups is important for providing more nuanced findings related to these patterns of vulnerability. Third‐level educational attainment was examined as an ordinal variable; however, not all young people will, or should, strive to obtain a university degree. A more insightful outcome measure for future research may be young people's satisfaction with their post‐school education choices or opportunities. Causal factors for SES differences in parental expectations and academic self‐concepts were not examined in the current study and could be an avenue for future research. Given the data available, we focus on two specific timepoints (9 and 13 years); however, latent class analysis of children's self‐concept development has highlighted the stability and predictive effects of initial scores as early as kindergarten (Musu‐Gillette et al., 2015). As such, efforts to mitigate against SES differences may be even more relevant in early childhood education and initial primary school years. Affirmative actions, such as the Higher Education Access Route (HEAR; 2024) scheme in Ireland at the point of higher education entry, can also support students experiencing socio‐economic inequality later in their education, which was not examined in the current study.

## CONCLUSIONS

This study draws on situated expectancy‐value theory (Eccles & Wigfield, 2020) and developmental systems theory (Lerner, 2006) to unpack how academic self‐concepts, parent expectations and teacher–child interactions contribute to SES differences in educational attainment. Bolstering children's academic self‐concepts prior to the secondary school transition, curbing steeper declines among economically vulnerable children and encouraging parent educational expectations could mitigate against persistent socio‐economic educational inequality. Teacher–child interactions moderate the link between parent expectations and educational attainment, but positive influences from both teachers and parents are needed to close attainment gaps among economically vulnerable students. These findings have utility for policy and practices targeting socio‐economic differences in educational attainment.

## AUTHOR CONTRIBUTIONS

**Caoimhe Dempsey:** Conceptualization; methodology; data curation; formal analysis; visualization; writing – original draft; writing – review and editing. **Selina McCoy:** Supervision; funding acquisition; writing – review and editing; project administration; resources.

## FUNDING INFORMATION

The authors gratefully acknowledge funding from the EU Horizon Program, project number Project 101061104 ESSPIN ‘ECONOMIC, SOCIAL AND SPATIAL INEQUALITIES IN EUROPE IN THE ERA OF GLOBAL MEGA‐TRENDS’. The opinions expressed in this document are the sole responsibility of the authors and do not necessarily represent the official position of the EU.

## CONFLICT OF INTEREST STATEMENT

The authors have no conflict of interest to declare.

## ETHICAL APPROVAL STATEMENT

Growing Up in Ireland was carried out under ethical approval granted by an independent Research Ethics Committee set up by the Irish Department of Children and Youth Affairs.

## Data Availability

The data that support the findings of this study are available from the Central Statistics Office. Restrictions apply to the availability of these data, which were used under licence for this study.

## Appendix

To identify the optimal number of latent classes that best fit the data, Maître et al. (2021) assessed model fit for models with one, two and three classes. Bayesian information criterion (BIC) and Akaike's information criterion (AIC) were used to assess model fit. The model with three classes produced the best fit; however, it was only marginally better than the two‐class model (see Table A2). The model with three classes produced respective class sizes of 80.4%, 14.8% and 4.8%. The small size of the third class limits its meaningful interpretation. Therefore, Maître et al. (2021) adopted the latent class model with two classes.

**TABLE A1:** Latent class profile of economic vulnerability and flows into and out of classes across the two timepoints.

		9 years (2007)	13 years (2011)
Size of EV group		9.2	28.5
Characteristics of EV group			
	Bottom income quintile	69.5	41.7
	Economic stress	59.9	78.8
	Material deprivation (mean deprivation of 11 items)	1.70	1.71
*N*		6039	6039
No. of EV		558	1719
Of whom			
	Stayers		388
	Inflow		1331
	Outflow		169

*Note*: Data in this table have been taken from Maître et al.'s (2021) original publication of this measure.

**TABLE A2:** Latent class model fit at 9 years (adapted from Maître et al., 2021).

Model	Observation	df	AIC	BIC
9 years				
One class	8563	4	17,458.89	17,487.12
Two classes	8563	8	16,210.34	16,266.78
Three classes	8563	12	16,194.07	16,278.74

## References

[bjep70025-bib-0001] Aiken, L. S. , West, S. G. , & Reno, R. R. (1991). Multiple regression: Testing and interpreting interactions. Sage.

[bjep70025-bib-0002] Ainsworth, M. D. S. , Blehar, M. C. , Waters, E. , & Wall, S. N. (2015). Patterns of attachment: A psychological study of the strange situation. Psychology Press.

[bjep70025-bib-0003] Aunola, K. , Leskinen, E. , Onatsu‐Arvilommi, T. , & Nurmi, J. E. (2002). Three methods for studying developmental change: A case of reading skills and self‐concept. British Journal of Educational Psychology, 72(3), 343–364. 10.1348/000709902320634447 12396310

[bjep70025-bib-0004] Bjorklund‐Young, A. (2016). Family income and the college completion gap . https://education.jhu.edu/edpolicy/commentary/collegegradgap

[bjep70025-bib-0101] Breen, R. , & Moisio, P. (2003). *Poverty dynamics for measurement error* (Vol. 17). ISER Working Papers. https://www.iser.essex.ac.uk/wp‐content/uploads/files/working‐papers/iser/2003‐17.pdf

[bjep70025-bib-0005] Briley, D. A. , Harden, K. P. , & Tucker‐Drob, E. M. (2014). Child char‐acteristics and parental educational expectations: Evidence for transmission with transaction. Developmental Psychology, 50, 2614–2632. 10.1037/a0038094 25285965 PMC4250430

[bjep70025-bib-0006] Brown, C. , & Putwain, D. W. (2022). Socio‐economic status, gender and achievement: The mediating role of expectancy and subjective task value. Educational Psychology, 42(6), 730–748. 10.1080/01443410.2021.1985083

[bjep70025-bib-0007] Brown, T. A. (2015). Confirmatory factor analysis for applied research. Guilford Publications.

[bjep70025-bib-0008] Buchmann, M. , Grütter, J. , & Zuffianò, A. (2022). Parental educational aspirations and children's academic self‐concept: Disentangling state and trait components on their dynamic interplay. Child Development, 93, 7–24. 10.1111/cdev.13645 34427921 PMC9290651

[bjep70025-bib-0009] Burger, K. , & Mortimer, J. T. (2021). Socioeconomic origin, future expectations, and educational achievement: A longitudinal three‐generation study of the persistence of family advantage. Developmental Psychology, 57(9), 1540–1558. 10.1037/dev0001238 34929097 PMC8694581

[bjep70025-bib-0010] Carroll, E. , McCoy, S. , & Mihut, G. (2022). Exploring cumulative disadvantage in early school leaving and planned post‐school pathways among those identified with special educational needs in Irish primary schools. British Educational Research Journal, 48(6), 1065–1082. 10.1002/berj.3815

[bjep70025-bib-0012] CASEL: Collaborative for Academic, Social, and Emotional Learning . (2020). CASEL's SEL Framework. https://casel.org/casel‐sel‐framework‐11‐2020/

[bjep70025-bib-0013] Coelho, V. A. , Marchante, M. , & Jimerson, S. R. (2017). Promoting a positive middle school transition: A randomized‐controlled treatment study examining self‐concept and self‐esteem. Journal of Youth and Adolescence, 46, 558–569. 10.1007/s10964-016-0510-6 27230119

[bjep70025-bib-0014] Crampton, A. , & Hall, J. (2017). Unpacking socio‐economic risks for reading and academic self‐concept in primary school: Differential effects and the role of the preschool home learning environment. British Journal of Educational Psychology, 87(3), 365–382. 10.1111/bjep.12154 28295189

[bjep70025-bib-0015] De Witte, K. , & François, M. (2023). Covid‐19 learning deficits in Europe: Analysis and practical recommendations: Analytical report. Publications Office of the European Union. https://data.europa.eu/doi/10.2766/881143

[bjep70025-bib-0096] Delaney, E. , McAteer, S. , Delaney, M. , McHugh, G. , & O'Neill, B. (2023). PIRLS 2021: Reading results for Ireland, Educational Research Centre. https://www.erc.ie/wp‐content/uploads/2023/05/PIRLS‐2021_Reading‐Results‐for‐Ireland.pdf

[bjep70025-bib-0016] Demetriou, A. , Kazi, S. , Makris, N. , & Spanoudis, G. (2020). Cognitive ability, cognitive self‐awareness, and school performance: From childhood to adolescence. Intelligence, 79, 101432. 10.1016/j.intell.2020.101432

[bjep70025-bib-0017] Dempsey, C. , Devine, R. , Fink, E. , & Hughes, C. (2023). Developmental links between well‐being, self‐concept and prosocial behaviour in early primary school. British Journal of Educational Psychology, 94, 425–440. 10.1111/bjep.12654 38114272

[bjep70025-bib-0018] Dempsey, C. , Devine, R. , Symonds, J. , Sloan, S. , & Hughes, C. (2024). Interacting adult‐child relationships and school adjustment: Findings from Growing Up in Ireland. Journal of Applied Developmental Psychology, 92, 101653. 10.1016/j.appdev.2024.101653

[bjep70025-bib-0019] Department of Education . (2017). DEIS plan 2017. Department of Education. https://www.gov.ie/en/policy‐information/4018ea‐deis‐delivering‐equality‐of‐opportunity‐in‐schools/#deis‐plan‐2017

[bjep70025-bib-0021] Department of Education . (2023b). Statement of strategy 2023–2025. Department of Education. https://www.gov.ie/en/publication/d7691‐department‐of‐education‐statement‐of‐strategy‐2023‐2025/

[bjep70025-bib-0106] Dishion, T. J. , & Patterson, G. R. (2016). The development and ecology of antisocial behavior: Linking etiology, prevention, and treatment. Developmental psychopathology, 29, 1–32.

[bjep70025-bib-0023] Duncan, G. J. , & Magnuson, K. (2012). Socioeconomic status and cognitive functioning: Moving from correlation to causation. Wiley Interdisciplinary Reviews: Cognitive Science, 3(3), 377–386. 10.1002/wcs.1176 26301469

[bjep70025-bib-0024] Duncan, G. J. , Ziol‐Guest, K. M. , & Kalil, A. (2010). Early‐childhood poverty and adult attainment, behaviour, and health. Child Development, 81(1), 306–325. 10.1111/j.1467-8624.2009.01396.x 20331669

[bjep70025-bib-0025] Eccles, J. S. (1993). School and family effects on the ontogeny of children's interests, self‐perceptions, and activity choices. Nebraska Symposium on Motivation. Nebraska Symposium on Motivation, 40, 145–208. 1340520

[bjep70025-bib-0026] Eccles, J. S. , & Wigfield, A. (1995). In the mind of the actor: The structure of adolescents' achievement task values and expectancy‐related beliefs. Personality and Social Psychology Bulletin, 21(3), 215–225. 10.1177/0146167295213003

[bjep70025-bib-0027] Eccles, J. S. , & Wigfield, A. (2020). From expectancy‐value theory to situated expectancy‐value theory: A developmental, social cognitive, and sociocultural perspective on motivation. Contemporary Educational Psychology, 61, 101859. 10.1016/j.cedpsych.2020.101859

[bjep70025-bib-0028] Eccles, J. S. , & Wigfield, A. (2024). The development, testing, and refinement of Eccles, Wigfield, and colleagues' situated expectancy‐value model of achievement performance and choice. Educational Psychology Review, 36(2), 51. 10.1007/s10648-024-09888-9

[bjep70025-bib-0029] Educational Research Centre . (2007). Drumcondra primary mathematics test – Revised. Educational Research Centre.

[bjep70025-bib-0030] European Anti‐Poverty Network (EAPN) . (2018). How is poverty measured? How is poverty measured? EAPN.

[bjep70025-bib-0031] Evans, G. W. , Li, D. , & Whipple, S. S. (2013). Cumulative risk and child development. Psychological Bulletin, 139(6), 1342–1396. 10.1037/a0031808 23566018

[bjep70025-bib-0032] Frenzel, A. C. , Goetz, T. , Pekrun, R. , & Watt, H. M. G. (2010). Developmentof mathematics interest in adolescence: Influences of gender, family, and school context. Journal of Research on Adolescence, 20, 507–537. 10.1111/j.1532-7795.2010.00645.x

[bjep70025-bib-0033] García, E. , & Weiss, E. (2017). Education inequalities at the school starting gate: Gaps, trends, and strategies to address them. Economic Policy Institute.

[bjep70025-bib-0034] Goodman, R. (1997). The strengths and difficulties questionnaire: A research note. Journal of Child Psychology and Psychiatry, 38(5), 581–586. 10.1111/j.1469-7610.1997.tb01545.x 9255702

[bjep70025-bib-0035] Guo, J. , Marsh, H. W. , Parker, P. D. , Morin, A. J. , & Yeung, A. S. (2015). Expectancy‐value in mathematics, gender and socioeconomic background as predictors of achievement and aspirations: A multi‐cohort study. Learning and Individual Differences, 37, 161–168. 10.1016/j.lindif.2015.01.008

[bjep70025-bib-0036] Gutman, L. M. , & Midgley, C. (2000). The role of protective factors in supporting the academic achievement of poor African American students during the middle school transition. Journal of Youth and Adolescence, 29(2), 223–249. 10.1023/A:1005108700243

[bjep70025-bib-0037] Hayes, J. , & Stewart, I. (2016). Comparing the effects of derived relational training and computer coding on intellectual potential in school‐age children. British Journal of Educational Psychology, 86(3), 397–411. 10.1111/bjep.12114 27062159

[bjep70025-bib-0040] Jacobs, J. E. , Lanza, S. , Osgood, D. W. , Eccles, J. S. , & Wigfield, A. (2002). Changes in children's self‐competence and values: Gender and domain differences across grades one through twelve. Child Development, 73(2), 509–527. 10.1111/1467-8624.00421 11949906

[bjep70025-bib-0041] Joseph, A. , Sylva, K. , Sammons, P. , & Siraj, I. (2024). Drivers of the socio‐economic disadvantage gap in England: Sequential pathways that include the home learning environment and self‐regulation as mediators. British Journal of Educational Psychology, 94(1), 22–40. 10.1111/bjep.12629 37527934

[bjep70025-bib-0042] Kievit, R. A. , Brandmaier, A. M. , Ziegler, G. , van Harmelen, A.‐L. , de Mooij, S. M. M. , Moutoussis, M. , Goodyer, I. M. , Bullmore, E. , Jones, P. B. , Fonagy, P. , Lindenberger, U. , & Dolan, R. J. (2018). Developmental cognitive neuroscience using latent change score models: A tutorial and applications. Developmental Cognitive Neuroscience, 33, 99–117. 10.1016/j.dcn.2017.11.007 29325701 PMC6614039

[bjep70025-bib-0043] Koshy, P. , Dockery, A. M. , & Seymour, R. (2019). Parental expectations for young people's participation in higher education in Australia. Studies in Higher Education, 44(2), 302–317. 10.1080/03075079.2017.1363730

[bjep70025-bib-0044] Kriegbaum, K. , & Spinath, B. (2016). Explaining social disparities in mathematical achievement: The role of motivation. European Journal of Personality, 30(1), 45–63. 10.1002/per.2042

[bjep70025-bib-0045] Leflot, G. , Van Lier, P. A. , Onghena, P. , & Colpin, H. (2010). The role of teacher behavior management in the development of disruptive behaviors: An intervention study with the good behavior game. Journal of Abnormal Child Psychology, 38, 869–882. 10.1007/s10802-010-9411-4 20373016

[bjep70025-bib-0046] Lerner, R. M. (2006). Resilience as an attribute of the developmental system: Comments on the papers of Professors Masten & Wachs. Annals of the New York Academy of Sciences, 1094(1), 40–51. 10.1196/annals.1376.005 17347340

[bjep70025-bib-0047] Maître, B. , Russell, H. , & Smyth, E. (2021). *The dynamics of child poverty in Ireland: Evidence from the Growing Up in Ireland survey* (No. 121). Research Series. https://www.esri.ie/system/files/publications/RS121.pdf

[bjep70025-bib-0048] Marsh, H. W. , & Martin, A. J. (2011). Academic self‐concept and academic achievement: Relations and causal ordering. British Journal of Educational Psychology, 81(1), 59–77. 10.1348/000709910X503501 21391964

[bjep70025-bib-0049] Marsh, H. W. , Pekrun, R. , Guo, J. , Hattie, J. , & Karin, E. (2023). Too much of a good thing might be bad: The double‐edged sword of parental aspirations and the adverse effects of aspiration‐expectation gaps. Educational Psychology Review, 35(2), 49. 10.1007/s10648-023-09768-8

[bjep70025-bib-0050] Martin, A. J. , Collie, R. J. , Stephan, M. , Flesken, A. , Halcrow, F. , & McCourt, B. (2024). The role of teaching support in assisting students' transition to high school. Learning and Individual Differences, 109, 102382. 10.1016/j.lindif.2023.102382

[bjep70025-bib-0051] Martin, A. J. , & Dowson, M. (2009). Interpersonal relationships, motivation, engagement, and achievement: Yields for theory, current issues, and educational practice. Review of Educational Research, 79(1), 327–365. 10.3102/0034654308325583

[bjep70025-bib-0052] Martins, J. , Cunha, J. , Lopes, S. , Moreira, T. , & Rosário, P. (2021). School engagement in elementary school: A systematic review of 35 years of research. Educational Psychology Review, 34, 793–849. 10.1007/s10648-021-09642-6

[bjep70025-bib-0053] Masten, A. S. , & Cicchetti, D. (2016). Resilience in development: Progress and transformation. In D. Cicchetti (Ed.), Developmental psychopathology: Risk, resilience, and intervention (pp. 271–333). John Wiley & Sons, Inc. 10.1002/9781119125556.devpsy406

[bjep70025-bib-0054] McCoy, S. , Maître, B. , Watson, D. , & Banks, J. (2016). The role of parental expectations in understanding social and academic well‐being among children with disabilities in Ireland. European Journal of Special Needs Education, 31(4), 535–552. 10.1080/08856257.2016.1199607

[bjep70025-bib-0100] McCoy, S. , Smyth, E. , Watson, D. , & Darmody, M. (2014). Leaving school in Ireland: A longitudinal study of post‐school transitions. ESRI Research Series, 36(1), 1–28. https://www.stratfordcollege.ie/content/archive/RS36_Leaving_School_In_Ireland__A_Longitudinal_Study_of_Post_School_Transitions_August_2014.pdf

[bjep70025-bib-0055] Michael, D. , & Kyriakides, L. (2023). Mediating effects of motivation and socioeconomic status on reading achievement: A secondary analysis of PISA 2018. Large‐Scale Assessments in Education, 11(1), 31. 10.1186/s40536-023-00181-9

[bjep70025-bib-0102] Moisio, P. (2004). A latent class application to the multidimensional measurement of poverty. Quality and Quantity, 38(6), 703–717. 10.1007/s11135-004-5940-7

[bjep70025-bib-0097] Mullis, I. V. , Martin, M. O. , Foy, P. , Kelly, D. L. , & Fishbein, B. (2020). TIMSS 2019 International Results in Mathematics and Science, TIMSS & PIRLS International Study Center, Lynch School of Education and Human Development, Boston College and International Association for the Evaluation of Educational Achievement.

[bjep70025-bib-0099] Murphy, D. , Quail, A. , Williams, J. , Gallagher, S. , Murray, A. , McNamara, E. , & O’Mahony, D. (2018). A summary guide to wave 3 of Growing Up in Ireland's Child cohort (at 17/18 years). Economic and Social Research Institute.

[bjep70025-bib-0056] Murray, A. (2022). Growing Up in Ireland: Key findings from the special COVID‐19 survey of Cohorts ‘98 and ‘08. Economic and Social Research Institute.

[bjep70025-bib-0057] Murray, A. , McRory, C. , Thornton, M. , Williams, J. , & Quail, A. (2011). Design, instrumentation and procedures for the child cohort. Growing Up in Ireland. https://www.growingup.gov.ie/pubs/BKMNEXT255.pdf

[bjep70025-bib-0058] Musu‐Gillette, L. E. , Wigfield, A. , Harring, J. R. , & Eccles, J. S. (2015). Trajectories of change in students' self‐concepts of ability and values in math and college major choice. Educational Research and Evaluation, 21(4), 343–370. 10.1080/13803611.2015.1057161

[bjep70025-bib-0059] Nauman, C. , Goble, P. , Alfaro, E. C. , & Weimer, A. A. (2023). Adolescent academic success: Teacher‐child interactions as a buffer for early childhood relational adversity. Journal of Child and Family Studies, 32(7), 1895–1910. 10.1007/s10826-022-02496-7

[bjep70025-bib-0060] OECD . (2023). PISA 2022 results. OECD. https://www.oecd.org/publication/pisa‐2022‐results/

[bjep70025-bib-0061] OECD . (2024). OECD review of resourcing schools to address educational disadvantage in Ireland, reviews of National Policies for education. OECD Publishing. 10.1787/3433784c-en

[bjep70025-bib-0062] Orth, U. , Dapp, L. C. , Erol, R. Y. , Krauss, S. , & Luciano, E. C. (2021). Development of domain‐specific self‐evaluations: A meta‐analysis of longitudinal studies. Journal of Personality and Social Psychology, 120(1), 145–172. 10.1037/pspp0000378 33252972

[bjep70025-bib-0063] Park, J. , Rose, J. , McKeown, S. , & Washbrook, E. (2024). Occupational aspirations and academic achievement: Rethinking the direction of effects and the role of socioeconomic status in middle childhood and adolescence. Journal of Social Issues, 80(4), 1408–1432. 10.1111/josi.12655

[bjep70025-bib-0064] Paulus, L. , Spinath, F. M. , & Hahn, E. (2021). How do educational inequalities develop? The role of socioeconomic status, cognitive ability, home environment, and self‐efficacy along the educational path. Intelligence, 86, 101528. 10.1016/j.intell.2021.101528

[bjep70025-bib-0065] Pekrun, R. (2021). Self‐appraisals and emotions: A generalized control‐value approach. In T. Dicke , F. Guay , H. W. Marsh , R. G. Craven , & D. M. McInerney (Eds.), Self—A multidisciplinary concept (pp. 1–30). Information Age Publishing.

[bjep70025-bib-0066] Perkins, R. , & Clerkin, A. (2020). TIMSS 2019: Ireland's results in mathematics and science. Educational Research Centre. https://www.erc.ie/wp‐content/uploads/2021/01/03‐ERC‐TIMSS‐2019‐Report_A4_Online.pdf

[bjep70025-bib-0068] Piers, E. V. , & Herzberg, D. S. (2002). Piers‐Harris 2: Piers‐Harris children's self‐concept scale. Western Psychological Services.

[bjep70025-bib-0070] Pinquart, M. , & Ebeling, M. (2020). Parental educational expectations and academic achievement in children and adolescents—A meta‐analysis. Educational Psychology Review, 32(2), 463–480. 10.1007/s10648-019-09506-z

[bjep70025-bib-0072] Postigo, Á. , Fernández‐Alonso, R. , Fonseca‐Pedrero, E. , González‐Nuevo, C. , & Muñiz, J. (2022). Academic self‐concept dramatically declines in secondary school: Personal and contextual determinants. International Journal of Environmental Research and Public Health, 19(5), 3010. 10.3390/ijerph19053010 35270703 PMC8910088

[bjep70025-bib-0073] R Core Team . (2022). R: A language and environment for statistical computing. R Foundation for Statistical Computing. https://www.R‐project.org/

[bjep70025-bib-0074] Ramos, A. , & Verschueren, K. (2024). Math self‐concept in the transition to secondary school: Developmental trends, predictors, and educational implications among high‐ability and average‐ability students. Journal of School Psychology, 103, 101268. 10.1016/j.jsp.2023.101268 38432723

[bjep70025-bib-0076] Rickert, N. P. , & Skinner, E. A. (2024). Parent and teacher involvement and adolescent academic engagement: Unique, mediated, and transactional effects. International Journal of Behavioral Development, 48(1), 71–84. 10.1177/01650254231210561

[bjep70025-bib-0077] Roorda, D. L. , Jak, S. , Zee, M. , Oort, F. J. , & Koomen, H. M. (2017). Affective teacher–student relationships and students' engagement and achievement: A meta‐analytic update and test of the mediating role of engagement. School Psychology Review, 46(3), 239–261. 10.17105/SPR-2017-0035.V46-3

[bjep70025-bib-0078] Sammons, P. , Hall, J. , Sylva, K. , Melhuish, E. , Siraj‐Blatchford, I. , & Taggart, B. (2013). Protecting the development of 5–11‐year‐olds from the impacts of early disadvantage: The role of primary school academic effectiveness. School Effectiveness and School Improvement, 24(2), 251–268. 10.1080/09243453.2012.749797

[bjep70025-bib-0079] Sammons, P. , Sylva, K. , Melhuish, E. C. , Siraj, I. , Taggart, B. , Elliot, K. , & Marsh, A. (2004). The effective provision of pre‐school education (EPPE) project: Technical paper 11—The continuing effects of pre‐school education at age 7 years. DfES/Institute of Education, University of London.

[bjep70025-bib-0080] Sammons, P. , Sylva, K. , Melhuish, E. C. , Siraj, I. , Taggart, B. , & Hunt, S. (2008). The effective pre‐school and primary education 3–11project (EPPE 3–11): Influences on children's attainment and progress in key stage 2: Cognitive outcomes in year 6. DCSF/Institute of Education, University of London.

[bjep70025-bib-0081] Satorra, A. , & Bentler, P. M. (2001). A scaled difference chi‐square test statistic for moment structure analysis. Psychometrika, 66(4), 507–514. 10.1007/BF02296192 PMC2905175 20640194

[bjep70025-bib-0083] Simpkins, S. D. , Fredricks, J. A. , Eccles, J. S. , & Huston, A. C. (2015). The role of parents in the ontogeny of achievement‐related motivation and behavioral choices. Monographs of the Society for Research in Child Development, 80, 1–169. https://www.jstor.org/stable/43773570 10.1111/mono.12156 25943024

[bjep70025-bib-0084] Smyth, E. (2020). Shaping educational expectations: The perspectives of 13‐year‐olds and their parents. Educational Review, 72(2), 173–195. 10.1080/00131911.2018.1492518

[bjep70025-bib-0085] Smyth, E. , & Privalko, I. (2023). The long road to secondary school: Background, home learning environment, and transition difficulties in Scotland. Research Papers in Education, 38(6), 847–864. 10.1080/02671522.2022.2065520

[bjep70025-bib-0086] Starr, C. R. , Ramos Carranza, P. , & Simpkins, S. D. (2022). Stability and changes in high school students' STEM career expectations: Variability based on STEM support and parent education. Journal of Adolescence, 94(6), 906–919. 10.1002/jad.12067 35754350

[bjep70025-bib-0087] Thornton, M. , Williams, J. , McCrory, C. , Murray, A. , & Quail, A. (2016). Design, instrumentation, and procedures for the child cohort at wave two (13 years). Growing Up in Ireland. https://www.growingup.gov.ie/pubs/BKMNEXT307.pdf

[bjep70025-bib-0088] Umarji, O. , McPartlan, P. , & Eccles, J. (2018). Patterns of math and English self‐concepts as motivation for college major selection. Contemporary Educational Psychology, 53, 146–158. 10.1016/j.cedpsych.2018.03.004

[bjep70025-bib-0089] Verschueren, K. , & Koomen, H. M. (2012). Teacher–child relationships from an attachment perspective. Attachment & Human Development, 14(3), 205–211. 10.1080/14616734.2012.672260 22537520

[bjep70025-bib-0090] Wang, M. T. , Degol, J. L. , & Henry, D. A. (2019). An integrative development‐in‐sociocultural‐context model for children's engagement in learning. American Psychologist, 74, 1086–1102. 10.1037/amp0000522 31829690

[bjep70025-bib-0103] Watson, D. , Maître, B. , Whelan, C. T. , & Williams, J. (2014). Dynamics of child economic vulnerability and socio‐emotional development: An analysis of the first two waves of the growing up in Ireland study. Department of Children and Youth Affairs, Dublin. http://www.dcya.gov.ie/docs/Dynamics_of_Child_Economic_Vulnerability_and_SocioEmotional_/3380.htm

[bjep70025-bib-0091] Whelan, C. T. , Watson, D. , Maître, B. , & Williams, J. (2015). Family economic vulnerability & the Great Recession: An analysis of the first two waves of the Growing Up in Ireland study. Longitudinal and Life Course Studies, 6(3), 230–244. 10.14301/llcs.v6i3.331

[bjep70025-bib-0092] Wigfield, A. , & Eccles, J. S. (2000). Expectancy–value theory of achievement motivation. Contemporary Educational Psychology, 25(1), 68–81. 10.1006/ceps.1999.1015 10620382

[bjep70025-bib-0093] Williams, J. , Greene, S. , Doyle, E. , Harris, E. , Layte, R. , McCoy, S. , McCrory, C. , Murray, A. , Nixon, E. , O'Dowd, T. , O'Moore, M. , Quail, A. , Smyth, E. , Swords, L. , & Thornton, M. (2009). Growing up in Ireland: The lives of 9‐year‐olds (Child Cohort Research Report No. 1). The Stationery Office.

[bjep70025-bib-0098] Williams, J. , Greene, S. , McNally, S. , Murray, A. , & Quail, A. (2010). Growing up in Ireland national longitudinal study of children. The infants and their families. The Stationery Office.

